# Controlling Protein Immobilization over Poly(3-hydroxybutyrate) Microparticles Using Substrate Binding Domain from PHA Depolymerase

**DOI:** 10.1021/acs.biomac.5c00010

**Published:** 2025-03-10

**Authors:** Isabela P. Dias, Regiane Stafim da Cunha, Ryu Masaki, Maritza A. Todo Bom, Edneia A. S. Ramos, Giovanna J. V. P. dos Santos, Giovanna Furman, Julia T. Lucena, Isabella G. Jiacomini, Sze M. Lo, Zelinda Schemczssen-Graeff, Breno C. B. Beirão, Silvio M. Zanata, Luiz M. de L. Faria, Edileusa M. Gerhardt, Emanuel Maltempi de Souza, Marcelo Müller-Santos, Guilherme F. Picheth

**Affiliations:** † Department of Biochemistry, Federal University of Paraná, Curitiba 80060-000, PR, Brazil; ‡ Department of Basic Pathology, Federal University of Paraná, Curitiba 80060-000, PR, Brazil; § Department of Genetics, Evolution, Microbiology and Immunology, University of Campinas, Campinas 13083-970, SP, Brazil; ∥ Department of Chemistry and Biology, Federal Technological University of Paraná, Curitiba 81531-980, PR, Brazil

## Abstract

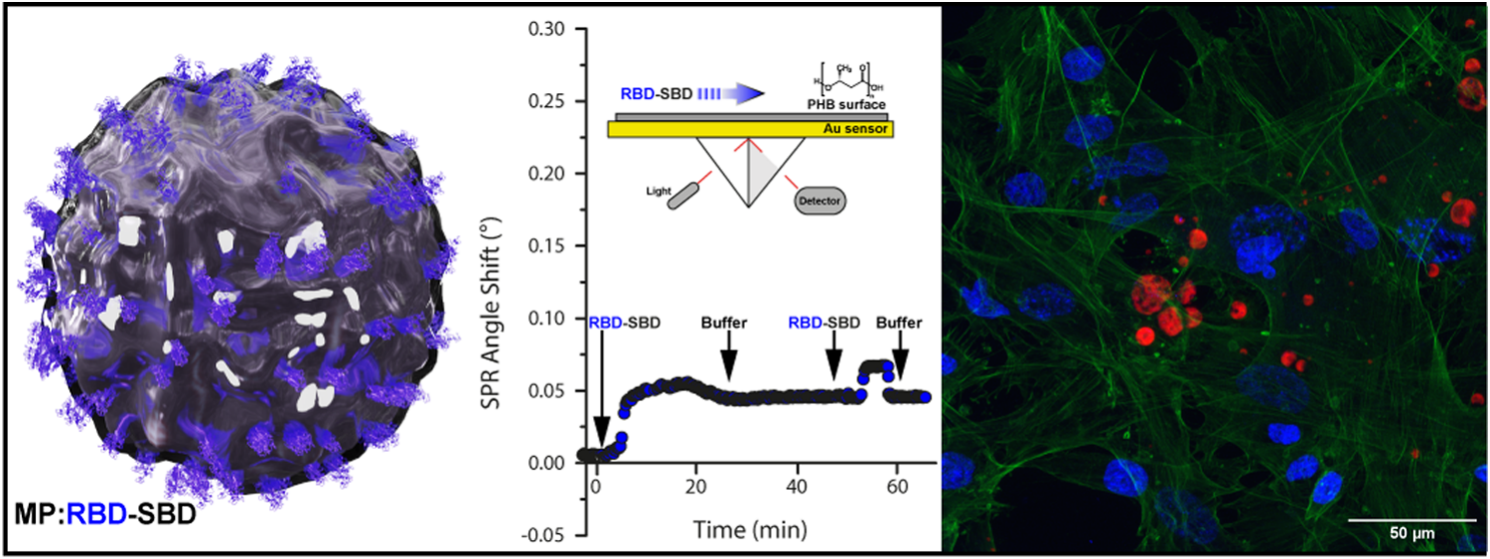

Biointerface decoration with ligands is a crucial requirement to modulate biodistribution, increase half-life, and provide navigation control for targeted micro- or nanostructured systems. To better control the process of ligand functionalization over three-dimensional (3D) polyester surfaces, we report the characterization of hybrid proteins developed to enhance the anchoring efficiency over polymeric surfaces and preserve optimal spatial orientation: sfGFP, mRFP1, and the RBD proteins were attached to a polyester substrate binding domain (SBD) formed by the C-terminus region of PHA depolymerase. The binding ability was evaluated over poly(3-hydroxybutyrate) (PHB) microparticles (MP) and two-dimensional (2D) surfaces. The PHB interfaces revealed a high affinity toward the proteins linked with SBD, displaying higher protein contents compared to untagged proteins. The MP decorated with RBD-SBD exhibited limited MRC5 internalization and cytotoxicity without a significant impact caused by the RBD protein, suggesting that the system might be adapted for targeted drug delivery and vaccine applications.

## 1 Introduction

Surface engineering is a near-interface modification strategy specifically designed to modulate interactions and tune the dynamics between different materials.^1^ This is a crucial concept in modeling synthetic biomaterials (e.g., scaffolds and delivery systems) projected to interact and generate specific responses from living systems. Since a myriad of cellular events are triggered via interfacial molecular recognition (e.g., energy transduction, transportation of matter), the surface properties of a particular material must be precisely adjusted for efficient stimuli-responsiveness and good biocompatibility.^2^

The successful clinical translation of advanced functional and bioactive biomaterials strictly depends on several surface features, such as hydrophobicity, surface charge, and stiffness.^3^ In particular, many applications require the immobilization of ligands (e.g., peptides, aptamers) along the biomaterial’s surface to induce particular and efficient biological responses.^4,5^ Accordingly, the ligand’s concentration, density, molecular arrangement, flexibility, conformation, and accessibility are key parameters for optimal cellular interactivity.^6^ However, most immobilization methods display poor control over ligand distribution across the interface, exhibit difficult stoichiometric regulation, and may modify the material itself, leading to undesired effects, such as steric hindrance, altered hydrodynamic diameter, or decreased stealth effect.^7^ In addition, the most common methodologies, such as adsorption or covalent conjugation, often cause ligand precipitation over the material’s surface and are inadequate to preserve the molecule’s dynamics or provide incorrect spatial orientation for receptor binding.^8−10^

Biointerface decoration with ligands is a crucial requirement for the physicochemical design of such materials intended for in vivo administration, for their ability to modulate the biodistribution, increase the half-life, and provide navigation control for targeted accumulation.^11^ Polyesters are among the most exploited biomaterials for the synthesis of drug delivery devices,^12^ vaccine vehicles,^13^ vascular embolization agents,^14^ hydrogels,^15^ and imaging probes.^16^ Such a wide range of applications is enabled by some attractive features of polyesters, such as the easiness of producing nano- or microstructures with good control over size, polydispersity, hydrophobicity, and *in vivo* biodegradability and biocompatibility.^13^ However, the processes of ligand surface functionalization over polyesters are not efficient and usually present (i) limited control over ligand density, (ii) difficult reaction control, (iii) multiple ligand organization/conformations, (iv) restricted flexibility across the interface, and (v) bioactivity loss.^17,18^

**Figure 1:**
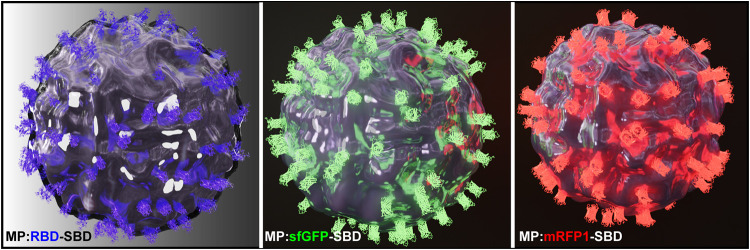
Schematic representation of PHB microparticles decorated with the hybrid proteins RBD-SBD, sfGFP-SBD, and mRFP1-SBD.

To better control the process of ligand functionalization and presentation over three-dimensional (3D) polyester surfaces, we report the characterization of a biotechnological strategy based on hybrid proteins comprising an active unit linked to a specific peptide sequence capable of anchoring over the polymeric surface and preserving optimal spatial orientation, as well as improve functionalization control. The emergence of bioinspired peptide sequences may expand the use of a wide variety of therapeutical and diagnostic devices, such as interchangeable vaccines or implantable materials.^19,20^ Here, we designed three different proteins formed by the bioactive units: superfolder green fluorescent protein (sfGFP), monomeric red fluorescent protein (mRFP1), and the receptor-binding domain (RBD) obtained from SARS-CoV-2 Spike protein, each attached to a polyester substrate binding domain (SBD) formed by the C-terminus region of PHA depolymerase from *Ralstonia pickettii*.^21^ The binding ability provided by SBD was evaluated over poly(3-hydroxybutyrate) (PHB) microparticles (Figure 1). This study provides useful information for the development of advanced ligand-mediated systems at the micro- or nanoscale.

## 2 Methodology

### 2.1 Materials

Poly(hydroxybutyrate) (PHB), poly(vinyl alcohol) (PVA, Mw 31 kDa, 88% hydrolyzed), sodium cholate (SC, >97.0%), Nile Red (microscopy grade), deuterated chloroform (CDCl_3_), 3-(4,5-dimethythiazol-2-yl)-2,5-diphenyltetrazolium bromide (MTT), MS-SAFE protease, and phosphatase inhibitors were obtained from Sigma-Aldrich. Fluoromount G with 4′-6-diamidino-2-phenylindole (DAPI) was purchased from Invitrogen (California). Ultrapure water was obtained using a Milli-Q purification system from Millipore. Fetal bovine serum (FBS), high-glucose Dulbecco’s modified Eagle’s medium (DMEM), trypsin-EDTA (0.05%), and phosphate-buffered saline (PBS) were purchased from Gibco (Waltham, MA). A Bio-Rad Protein Assay kit was purchased from Bio-Rad (Hercules, CA). All other reagents were commercial products of the highest available purity grade.

### 2.2 Protein Expression Vector Construction

The SARS-CoV-2 Spike RBD domain sequence (amino acids between 319 and 541) was retrieved from the genome of the isolated Wuhan-Hu-1 (Genbank accession, NC_045512.2.; S1 protein ID, YP_009724390). The PHA depolymerase SBD domain sequence of *Ralstonia pickettii* T1 (formerly known as *A. faecalis* T1), comprising the amino acids from 424 to 488, was extracted from the sequence deposited in the Genbank (Protein ID, AAA21974.1).^22^ An artificial construct, named 6-His-RBD-SBD, was designed to contain the RBD coding sequence fused to 6-histidines at the N-terminal portion and to the SBD at the C-terminal coding sequences. The sequence was codon-optimized for *Escherichia coli* and synthesized by Twist Bioscience (San Francisco, California, EUA). This synthetic gene was cloned into pET29b(+) as a NdeI and XhoI fragment to produce plasmid pET29b-RBD-SBD.

For the HEK293 F expression of RBD-SBD, it was cloned into pCDNA 3.1(+) between T7 and His-tag sequences. The construct contained the IL-2 signal peptide (AAB86861.1) for protein secretion. The final construct for mammalian expression was the plasmid IL-2 signal, IL-2 signal-RBD-SBD-6-His.

To construct recombinant plasmids expressing the proteins sfGFP-SBD and mRFP1-SBD, PCR fragments containing the coding sequences for sfGFP (superfolder green fluorescent protein) and mRFP1 (monomeric red fluorescent protein) were amplified from plasmids BBa_I746908 and J61002 (iGEM repository), respectively. The SBD coding sequence was amplified from pET29b-RBD-SBD. The resulting fragments were subcloned into the pBluescript KS(+) vector, confirmed by sequencing, and assembled into the pET28a(+) vector between the NcoI and XhoI sites using the Golden Gate method^23^ to yield pET28a-sfGFP-SBD and pET28a-mRFP1-SBD, expressing sfGFP-SBD and mRFP1-SBD proteins, respectively.

### 2.3 Overexpression of Recombinant Proteins

*E. coli* BL21(λDE3) carrying the expression vectors was cultivated in 80 mL of LB medium in 250 mL Erlenmeyer flasks at 30 °C for 2 h. After the autoinduction protocol, the culture was centrifuged at 5000*g* at 4 °C for 10 min. The supernatant was discarded, and the pellet was resuspended in Buffer A (50 mM Tris-HCl) pH 8.0, 150 mM NaCl, and sonicated during 10 cycles of 10 s (10 s ON/OFF) on an ice bath. The lysate was then centrifuged (12,000*g*, 4 °C, 10 min); the soluble fraction was discarded. RBD-SBD and mRFP1-SBD proteins were expressed as inclusion bodies that required solubilization and refolding.

HEK293 F cells were cultured in suspension and were used at 2 × 10^6^ cells/mL in FreeStyle 293 medium without antibiotics. Cells were transfected using poly(ethylene imine) (PEI) (Polysciences) in a 3:1 ratio with the pCDNA 3.1 plasmid (v/m). Reagents were diluted in Opti-MEM reduced serum medium before transfection. After 24 h, FreeStyle 293 medium with antibiotics was added to the cells in a 1:1 ratio. After 5–6 days of expression, the cells were centrifuged, and the supernatant was kept in a freezer at −80 °C until purification.

#### 2.3.1 Purification of RBD-SBD

RBD-SBD from bacteria were obtained from inclusion bodies (IBs). The IBs were washed three times with 1 mL of Buffer B (50 mM Tris-HCl at pH 8.0, 0.5% Triton X-100, 1 M urea). Following the washes, the IBs were solubilized in 1 mL of 8 M urea at room temperature, pipetting up and down 10 times. The solution was not wholly translucent after solubilization with urea; however, after centrifugation (12,000*g*, 4 °C, 10 min), there was enough soluble RBD-SBD to proceed to the refolding step.

One mL of urea-solubilized protein was transferred to a 12 kDa-cutoff dialysis cellulose membrane tube and dialyzed against 1 L of Buffer C (50 mM Tris-HCl pH 9.0, 150 mM NaCl) for 24 h at room temperature. The dialyzed protein solution was centrifuged (12,000*g*, 4 °C, 10 min), and the supernatant was injected at a flow rate of 1 mL min^–1^ into a 1 mL HiTrap Chelating Ni^2+^-charged (Thermo Fisher Scientific) column equilibrated with Buffer D (50 mM Tris-HCl pH 9.0, 150 mM NaCl, 5% glycerol, 20 mM imidazole). The column was washed with 12 column volumes of Buffer D. The RBD-SBD was eluted in Buffer E (50 mM Tris-HCl) (pH 9.0, 150 mM NaCl, 200 mM imidazole). Imidazole was removed by dialysis against 2 L of Buffer C for 24 h at room temperature.

HEK293 F-derived RBD-SBD was purified from the culture supernatants. Purification by affinity chromatography was performed using a 5 mL Histrap HP or HiTrap Chelating HP in the ÄKTA purification system (Cytiva) using an increasing imidazole gradient between 10 and 500 mM for washing and protein elution (20 mM NaH_2_PO_4_, 500 mM NaCl and imidazole, pH 8.0).

#### 2.3.2 Purification and Refolding of Recombinant Protein mRFP1-SBD

Inclusion bodies were separated from the soluble fraction by centrifugation and resuspended in 8 mL of Buffer B (50 mM Tris-HCl pH 8.0, 0.5% Triton X-100, 1 M urea), followed by centrifugation at 4 °C and 20,000*g* for 5 min; this procedure was repeated three times. After the last centrifugation, inclusion bodies were resuspended in 8 mL of buffer C (50 mM Tris-HCl pH 8.0, 8 M urea) at room temperature and transferred to a cellulose dialysis tubing (12 kDa MWCO), then dialyzed against 2 L of Buffer A for 24 h. The protein was then purified through gel-filtration using a Sephadex G-50 (Sigma-Aldrich) column, using Buffer A as eluent.

#### 2.3.3 Purification of Recombinant Protein sfGFP-SBD

Cell pellets were resuspended in Buffer A (50 mM Tris-HCl pH 8.0, 150 mM NaCl) at one-tenth of culture volume, sonicated 15 times (10 s on/off) in an ice bath, and then centrifuged (20,000*g*, 4 °C, 20 min). The soluble fraction was then injected at a flow rate of 1 mL min^–1^ in a HiTrap Chelating Ni^2+^-charged (Thermo Fisher Scientific) column charged with 100 mM NiCl_2_ and equilibrated with Buffer B. The protein was eluted with Buffer C (50 mM Tris-HCl, pH 8.0, 150 mM NaCl, 200 mM Imidazole) and dialyzed against 2 L of Buffer A overnight with a cellulose dialysis tubing (12 kDa MWCO).

#### 2.3.4 SDS-PAGE Analysis

The purity of the recombinant proteins was analyzed by SDS-PAGE in 12% SDS-polyacrilamide gels under reducing conditions.^24^ The molecular weight was estimated by using a protein marker. The gel was stained with Coomassie blue R-250 or by silver nitrate staining.^25^ The proteins were quantified using the Bradford method.^26^

### 2.4 Microparticle Formulation

Microparticles were prepared using the emulsion-evaporation technique.^27^ In brief, 25 mg of PHB was dissolved in 2 mL of chloroform in a 50 mL glass flask. Subsequently, 10 mL of poly(vinyl alcohol) (PVA) solution (1% w/v) was added, and the flask was tightly sealed with a rubber cap. The mixture was then subjected to high-speed homogenization for 1 min using a maximum speed (Gehaka, AV-2) to ensure thorough mixing. The solvent was gradually evaporated under slow and constant magnetic stirring (300 rpm) at room temperature (25 °C) until a homogeneous microsphere suspension was obtained. Some samples were also prepared with 100 μL of Nile Red (0.62 mg mL^–1^) that was added during polymer dissolution in chloroform to allow polymer visualization under fluorescence imaging.

### 2.5 Protein Immobilization

A dispersion of 0.1 mg mL^–1^ of PHB microparticles in 50 mM Tris-HCl and 150 mM NaCl buffer (pH 8.0) was used for the immobilization assay. The protein binding capacity on the microparticle surface was evaluated in five cycles, using increasing protein concentrations (15, 35, 50, 65, and 85 μg), with each incubation lasting 15 min. After each immobilization cycle, the microparticles were centrifuged at 3500*g* for 3 min at 15 °C. The proteins in the supernatant after immobilization were quantified using the Bradford method. The quantity of immobilized protein was deduced from the protein offered and the remaining proteins in the supernatant.

### 2.6 Scanning Electron Microscopy (SEM)

The surface and cross-sectional morphologies of the microparticles were investigated by scanning electron microscopy (SEM, Tescan Vega3 LMU). Microparticles were deposited on a mica slide, placed on carbon conductive double-sided adhesive tape, and dried at room temperature, which were mounted on specimen holders and then coated with gold in a sputter coater SCD030 (Balzers Union, FL 9496). After gold coating, the images were captured using an accelerating voltage of 15 kV. An X-ray detector coupled to SEM under the same operating conditions was used to take the electron micrographs.

### 2.7 Atomic Force Microscopy (AFM)

Topography images were obtained in a MultiMode8 atomic force microscope (Bruker) in peak force tapping mode. A SCANASYST-AIR probe (Bruker) with a spring constant of 0.4 N m^–1^ and a 2.0 nm tip-end radius was used for the scanning. All samples were diluted in ultrapure water, and 50 μL was deposited over a mica substrate and dried under a gentle N_2_ flow for 3 h. The AFM platform was mounted in an environmental chamber, which allows control over the relative humidity and the temperature (10% and 25 °C).

### 2.8 Confocal Laser Scanning Microscopy

For microscopy analysis, the sample suspensions were prepared by mixing 10 μL of microparticles with the same volume of glycerin and placing them between glass slides. The MRC5 cells were plated under round coverslips in a 24-well plate at a density of 10^5^ cells per well. After 18 h, the cells were treated with microparticles at 100 μg.mL^–1^ for 3, 6, 12, and 24 h. Next, the cells were washed with PBS, fixed with paraformaldehyde 2% (w/v), washed again with PBS, and the nucleus was stained with Fluoromount G with 4′-6-diamidino-2-phenylindole (DAPI) and the cytoskeleton with actin-green (Invitrogen). Laser scanning confocal, multiphoton microscope, model A1MP + (Nikon, Japan) at the Center for Advanced Fluorescence Technologies (CTAF-UFPR), using a 60× objective (NA 1.40, oil immersion), examined the slides. Fluorescence imaging was acquired, exciting Nile Red at 561 nm and capturing the light emission with the filter 595/50 (570–620 nm bandpass). Stacks of images were collected every 0.5 μm along the *z*-axis. All images were captured with the software Nis Elements 4.20 (Nikon, Japan), and measurements were processed using ImageJ software v1.51n.

### 2.9 Surface Plasmon Resonance

To evaluate protein binding, PHB 2D surfaces were assembled onto gold-coated surface plasmon resonance (SPR) sensors (BioNavis, Finland). In brief, a PHB solution was prepared in chloroform at 0.2 mg mL^–1^, and 20 μL were deposited on the sensor surface and allowed to dry for 2 h at room temperature inside the hood. Afterward, the sensors were washed with abundant ultrapure water for 2 h and allowed to dry. The sensors were placed inside the equipment and exposed to a continuous 20 μL min^–1^ flow of 50 mM Tris-HCl buffer pH 8 at 20 °C. The interaction with RBD-SBD was analyzed in real-time by injecting 100 μL of protein solution with a flow of 20 μL min^–1^ at 20 °C. The observed shifts were recorded after the rinsing step with 50 mM Tris-HCl buffer pH 8.

### 2.10 Quartz Crystal Microbalance

The PHB binding affinity with sfGFP-SBD and mRF1-SBD was also analyzed by quartz crystal microbalance (QCM) experiments. A PHB surface was deposited over HC49U silver-coated piezoelectric sensors by adding 20 μL of PHB dissolved in chloroform at 0.2 mg mL^–1^. After solvent evaporation for 2 h, the sensors were mounted inside a homemade QCM built with an OpenQCM shield and an Arduino microcontroller. The frequency shits were recorded after exposing the surface to 20 μL of the proteins for 30 min and washed with 50 mM Tris-HCl buffer pH 8 for an additional 30 min. The mass of absorbed PHB and protein was calculated using eq 1
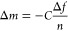
1where Δ*m* is the mass change, *C* is the mass sensitivity constant, Δ*f* is the change in resonance frequency, and *n* is the harmonic number.

### 2.11 Cytotoxicity Assay

To assess the *in vitro* cytotoxicity of the PHB MPs, the MTT assay that evaluates the mitochondrial activity was employed.^28^ MRC5 cells were plated in a 96-well culture plate (3 × 10^3^ cells/well). After 18 h, the cells were treated with microparticles at concentrations of 5, 10, 50, 100, and 500 μg mL^–1^ for 3, 6, 12, and 24 h. Subsequently, the medium was exchanged for fresh medium (90 μL/well) and 10 μL/well of the MTT solution (5 mg mL^–1^) and incubated for 3 h at 37 °C and 5% CO_2_ (Sigma, St. Louis). Finally, the medium was discarded, and 100 μL/well of dimethyl sulfoxide (DMSO) (Sigma, St. Louis) was added to dissolve the formazan crystals. The absorbance [*A*] was measured with a microplate reader at 570 nm, and the cell viability was calculated according to eq 2
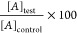
2where [*A*]_control_ corresponds to the absorbance of untreated control cells.

### 2.12 Flow Cytometry Analysis

The MPs were labeled with Nile Red during the formulation step for flow cytometry assays. After protein immobilization and centrifugation, they were exposed to the cells for 24 h. Afterward, the cells were detached from the flasks and fixed with 2% paraformaldehyde (PFA) for 20 min. The samples were analyzed in a FACSCalibur instrument (BD Biosciences). The intracellular fluorescence data was acquired using a 635 nm red diode laser and a 670 nm bandpass filter (FL4-H) controlled by CellQuest Pro software. In the cytometer, 10,000 events of each sample were acquired, and the data was analyzed and transformed into percentages, considering treated samples to the controls.

### 2.13 Western Blotting Analysis

Cells were lysed in RIPA buffer (150 mM sodium chloride, 1.0% Triton X-100, 0.5% sodium deoxycholate, 0.1% sodium dodecyl sulfate, and 50 mM Tris-HCl, pH 8.0), supplemented with MS-SAFE protease and phosphatase inhibitors. The samples were centrifuged at 10,000*g*, and the supernatant was collected. Protein concentration was determined by using the Bio-Rad Protein Assay kit. The proteins (50 and 70 μg) were heated at 95 °C for 10 min and loaded onto 8% SDS-polyacrylamide gel for electrophoresis, followed by a transfer to a nitrocellulose membrane (GE Healthcare, U.K.). The membranes were blocked for 1 h with TBS containing 0.05% Tween 20 and 5% BSA. After that, the membranes were incubated for 1 h in a Tris-buffered saline (TBS) solution containing 0.05% Tween 20 and 5% bovine serum albumin (BSA). Subsequently, the membranes were incubated with anti-ACE2 (1:1000, #92485, Cell Signaling Technology) and antiactin (#MA5–11869, Invitrogen) antibodies for 18 h at 4 °C. This was followed by a 1 h incubation with antimouse and antirabbit secondary antibodies at room temperature (19–23 °C). Immunolabeling was detected by chemiluminescence.

### 2.14 Statistical Analysis

The experiments on cell lines were repeated at least three times to ensure reliable results (biological replicates). The means and standard deviations were calculated using GraphPad Prism 6.0 software. ANOVA, or Student’s *t* test, was employed to assess the significance of the data. The statistical significance levels were set at *p* < 0.05 (*), *p* < 0.01 (**), and *p* < 0.001 (***), indicating the probability of obtaining such results by chance.

## 3 Results and Discussion

### 3.1 Protein Characterization

All of the model surfaces assembled as microparticles were prepared using PHB, a short-chain poly(hydroxyalkanoate) (PHA) polymer synthesized by several bacteria and archaea as an energy reserve material.^29^ PHB is a biocompatible and biodegradable polyester extensively utilized in many biomedical applications, such as scaffolds, vaccines, and drug delivery systems.^13,30,31^ As PHB is synthesized, it accumulates as 100–500 nm beads at the cytoplasm of microorganisms and is dynamically stabilized by proteins (e.g., PHA synthase, PHA depolymerase, and phasins).^32^ These specific classes of proteins exhibit amphiphilic character and can bind and modulate the PHB bead interface during the polymerization and depolymerization processes.^33^ In particular, the PHA depolymerase is structurally characterized by a lipase-like catalytic domain linked to a substrate binding domain (SBD), which is responsible for the enzyme adsorption over the polymer surface via hydrophobic interactions.^34^ The SBD attachment over water-insoluble substrates may inspire the design of a new bioengineered generation of targeting moieties specially modified to bind in a controlled manner over nanostructured materials. Therefore, we analyzed the binding ability of SBD and the bioactivity preservation of different ligands over a microstructured model of PHB particles.

All proteins were characterized by SDS-PAGE (Figure S1), which revealed distinct electrophoretic profiles based on the presence of SBD (between 30 and 40 kDa). The unmodified samples (mRFP1 and sfGFP) displayed a molecular weight of ∼30 kDa in SDS-PAGE, whereas those containing the SBD sequence exhibited reduced electrophoretic mobility (RBD-SBD, mRFP1-SBD, and sfGFP-SBD). The mass increment may be attributed to the SBD domain, with an expected *M*_w_ ∼ 10 kDa for the *R. pickettii* protein,^35^ indicating that the SBD domain was effectively introduced at the protein structures.

### 3.2 Microparticle Characterization and Ligand Immobilization

**Figure 2:**
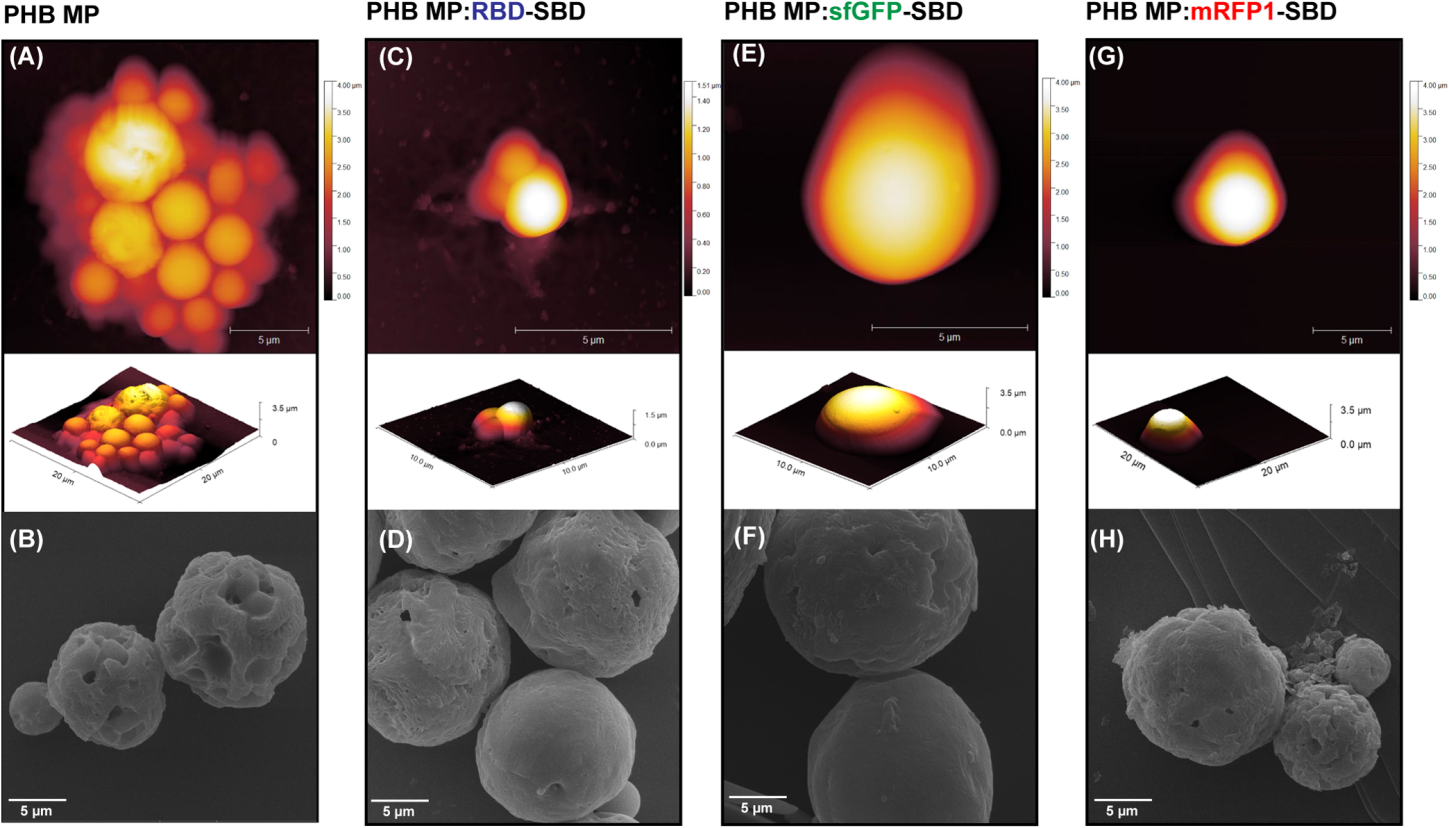
PHB microparticle characterization. 2D and 3D topographical AFM images of microparticles prepared in the absence (A) and after 5 cycles of ligand immobilization (C, E, G). MEV images of microparticles prepared in the absence (B) and after 5 cycles of ligand immobilization (D, F, H).

The PHB was initially formulated into microparticles (MP) of 10 ± 2 μm by the emulsification-evaporation method in the presence of PVA.^36^ The samples displayed spherical morphology containing pores and uneven edges (Figure 2A–C), probably as a result of the polymer/surfactant interfacial tension effect during shear stress and the fast process employed.^37^ The MP submitted to protein immobilization displayed the same morphology and size parameters as the control group, indicating that the ligands cause no effect on the overall surface structure (e.g., structure disruption or compression; Figure 2C–H).

**Figure 3:**
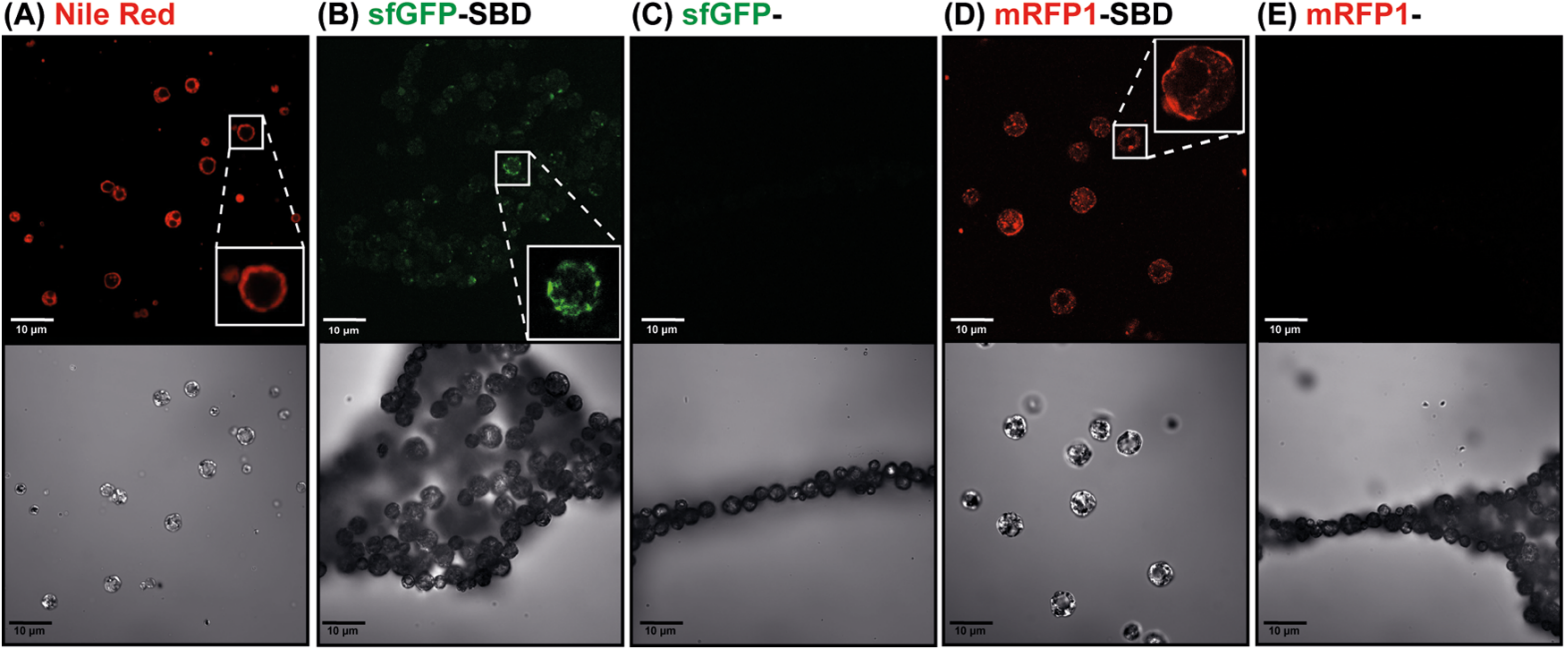
Confocal microscopy images of microparticles containing Nile red (A), sfGFP-SBD (B), sfGFP- (C), mRFP1-SBD (D), and mRFP1- (E) after 5 protein immobilization cycles.

To confirm protein immobilization and fluorescence activity, the MP exposed to the different ligands was analyzed by CLSM. Since the PHB MP displays no fluorescence, a Nile Red-containing sample was prepared to analyze the morphology. As shown in Figure 3A, most of the MP is hollow and exhibits irregular edges in accordance with SEM and AFM data. After incubation with the sfGFP-SBD and mRFP1-SBD proteins, all samples displayed fluorescence intensity localized at the MP interface in a concentration-dependent manner (Figure 3B,D). It is possible to observe that the proteins maintain their activity and are randomly organized over the surface, corroborating the diffuse fluorescence signal compared to Nile Red. However, the proteins synthesized without the SBD domain (sfGFP- and mRFP1-) revealed almost no fluorescence intensity after 5 immobilization cycles (Figure 3C,E), suggesting that the effective immobilization of these proteins is strictly dependent on the SBD peptide.

**Figure 4:**
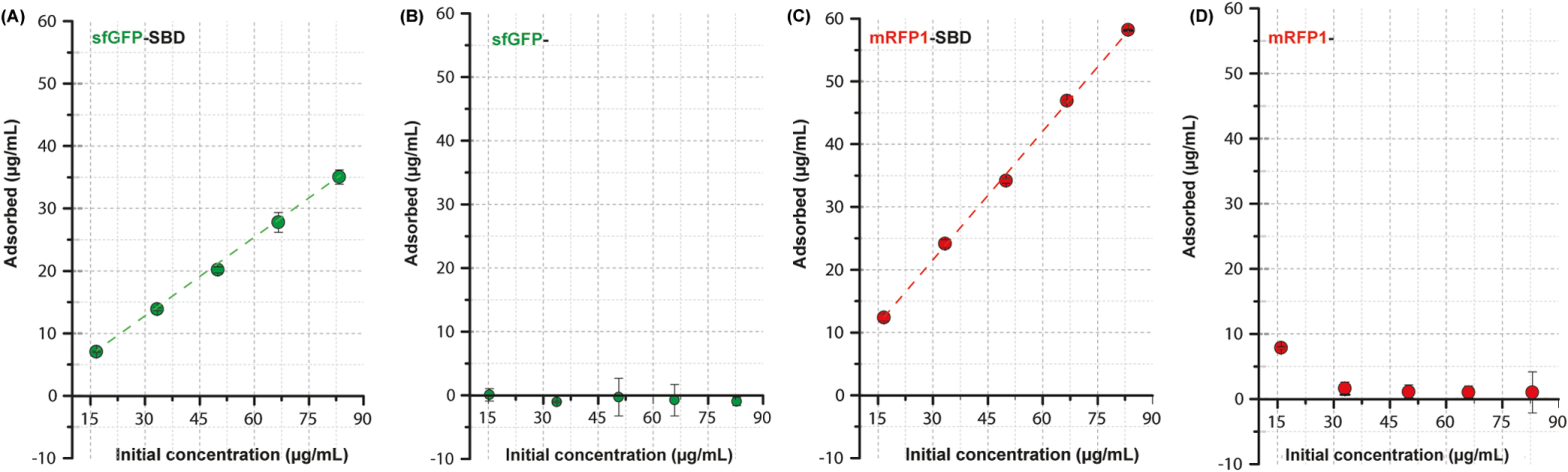
Protein adsorption quantification over the microparticles via fluorimetry for sfGFP-SBD (A), sfGFP- (B), mRFP1-SBD (C), and mRFP1- (D).

The MP’s protein immobilization performance was evaluated by quantitative fluorometric analysis as shown in Figure 4 and via Bradford assay in Figure S2. By maintaining a fixed MP concentration (0.1 mg mL^–1^) and increasing the protein amount (15–90 μg/mL) in independent vials, we observed that only the samples containing the SBD domain adsorbed at the PHB interface, in accordance with CLSM data. Thereby, the increment on sfGFP-SBD, mRFP1-SBD, and RBD-SBD protein concentrations leads to a linear increase in immobilized protein, either via fluorimetry or Bradford analysis, whereas sfGFP- and mRFP1- showed limited adsorption. Likewise, the RBD-SBD samples displayed a similar profile compared to the fluorescent proteins (Figure S2E), confirming the requirement of the SBD unit for irreversible immobilization. However, the binding efficiency is ∼50% for both sfGFP-SBD and RFP-SBD for most of the concentration range analyzed. These results suggest that a significant fraction of the protein remains unbound, likely due to protein autoassociation. This self-association may sequester the SBD domain within sfGFP or mRFP1 oligomers, hindering its ability to attach and interact effectively with the PHB interface. Notably, all untagged proteins show no sign of aggregation at the same concentration range,^38,39^ indicating a self-assembly influence induced by the SBD domain that may impact overall adsorption.

**Figure 5:**
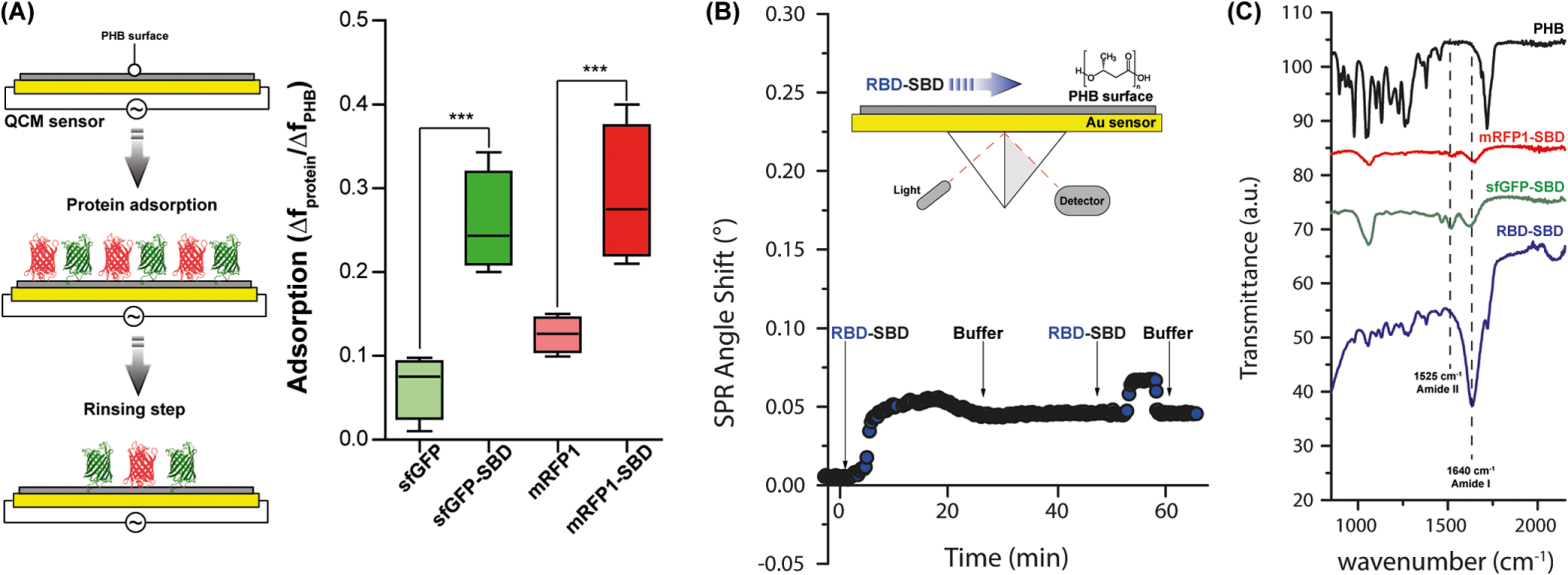
Schematical representation of QCM analysis procedure and adsorption data for sfGFP-, sfGFP-SBD, mRFP1, and mRFP1-SBD after the rinsing step (A). SPR real-time analysis of RBD-SBD binding over PHB 2D surfaces (B) and FTIR spectra of surfaces after protein adsorption and extensive rinsing (C).

To further investigate the interaction between PHB and the SBD-tagged proteins, we assembled a polymeric 2D model surface on silver-coated sensors to perform QCM and SPR experiments. The QCM results are represented as a ratio between the frequency variation for deposited PHB and the adsorbed protein after the respective rinsing step (Figure 5A): after PHB deposition, the sensors were exposed to a gentle flow of protein solution and were subsequently thoroughly rinsed with Tris-HCl buffer. The QCM assay exhibited limited adsorption of the untagged proteins sfGFP- and mRFP1-, whereas sfGFP-SBD and mRFP1-SBD showed a >2-fold signal increase compared to their counterparts. The adsorption profile of RBD-SBD was analyzed in multiple steps by SPR (Figure 5B), revealing a significant angle shift that corroborates the attraction toward the PHB surface: only a small amount of protein was removed during the rising step, probably as a result of unspecific interactions with the surface caused by the local adsorbate excess. However, another protein injection into the system causes no angle shift after the following rinsing step, indicating that the surface was already saturated. At the end of the processes, all sensors were analyzed by FTIR (Figure 5C), which showed specific SBD-tagged protein vibrations for all samples onto the PHB, characterized by the amide I at 1640 cm^–1^ and amide II 1525 cm^–1^ vibrations.^40^ These results suggest more efficient and irreversible protein adsorption mediated by the SBD domain.

### 3.3 PHB MP Decorated with RBD-SBD *In Vitro* Effects

**Figure 6:**
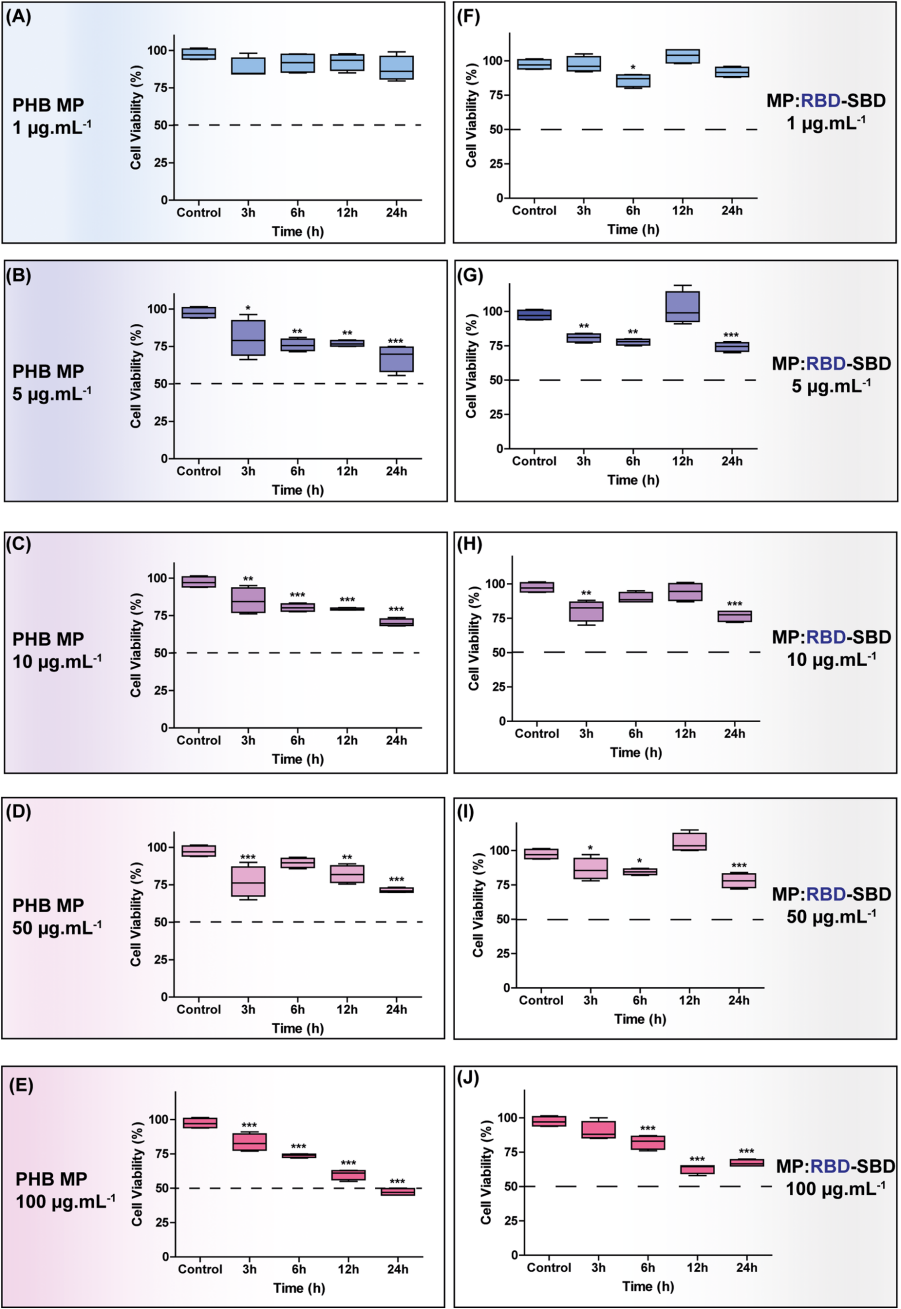
MRC5 cellular viability after exposure to PHB MP. MTT time-dependent assay for MRC5 incubated with uncoated MP at different concentrations (A–E). MTT time-dependent assay for MRC5 incubated with MP decorated with RBD-SBD at different concentrations (F–J).

Although PHB is considered a biocompatible and biodegradable polymer,^41^ specific surface modifications may alter the biodistribution of structured materials and, to an extent, its toxicological profile. To determine the impact of ligand immobilization over the cytotoxicity of MP, MCR5 cell lines, human–derived lung fibroblast cells, were utilized as a model for samples decorated with RBD-SBD. The MP (with or without RBD-SBD) were exposed to MCR5, and cell viability was analyzed via MTT in different concentrations (1–100 μg mL^–1^) and incubation periods (3–24 h). The MP devoid of protein decoration significantly reduced cell viability only when the cells were exposed to >5 μg mL^–1^ of MP for more than 6 h (Figure 6A–E). A similar profile was observed for the MP containing 8.75 μg of RBD-SBD, where the 100 μg mL^–1^ samples provoked greater cell viability reduction in 6, 12, and 24 h (Figure 6F–J).

As a result, the presence of RBD-SBD caused limited influence on the cytotoxicity studies, suggesting identical uptake and metabolic pathways. The cytotoxic effect observed for 5–100 μg mL^–1^ MP with or without RBD-SBD may be attributed to phagocytosis or nonspecific endocytosis, followed by lysosomal membrane destabilization and hyperosmolarity caused by concentration increment. This effect possibly leads to apoptotic mechanisms, reactive oxygen species generation, and DNA damage.^42^ As a result, the increment of MP accumulation might overcome the cell metabolic capacity, even at low concentrations and short time intervals, and induce cytotoxicity. In fact, increased intracellular amounts of biopolymers might be related to cytotoxic events, particularly when they are formulated at the microscale and exhibit high *M*_w_ and melting temperature (*T*_m_), which is the case for the PHB MP (∼10 μm, *M*_w_ ∼ 640.000 g mol^–1^ and *T*_m_ ∼ 172–180 °C). All of these characteristics are associated with decreased micro-Brownian motion and, consequently, enzymatic efficiency.^43^ Similar outcomes are observed for related biopolymers, such as poly(ε-caprolactone) and chitosan.^44^

**Figure 7:**
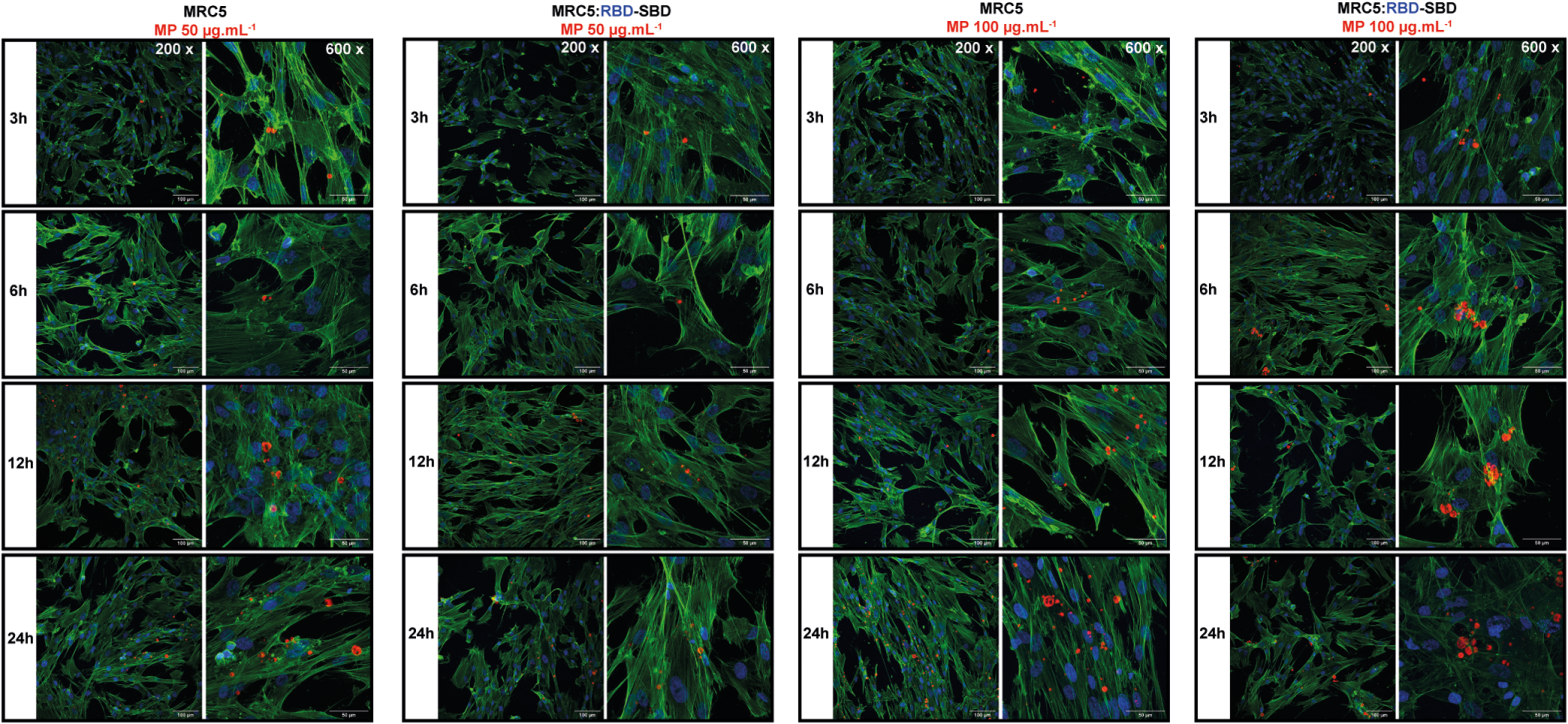
CLSM images of MRC5 cells incubated with MP (100 μg mL^–1^) decorated with RBD-SBD in different time intervals.

To confirm the hypothesis of MP internalization and correlate it with the cytotoxicity, we formulated samples containing the fluorescent probe Nile Red and followed the system accumulation and MRC5 morphology in different time intervals by CLSM. At MP concentration of 5 μg mL^–1^, it is possible to observe a discrete amount of samples inside the cells only after 3 h (indicated by the white arrows in Figure S3A–D), whereas at 0.5 and 1 h, no fluorescent signal is detected. At the MP concentration of 50 and 100 μg mL^–1^, a significant increment in MPs is observed within the cell cytoplasm, which is remarkably greater for the 100 μg mL^–1^ samples: from 3–24 h, the amount of polymer increases, and it is possible to distinguish up to 10 μm MP, as well as fully or partly degraded samples inside the cells (Figure 7). Along with MP internalization, the images exhibit a significant modification in cell morphology after incubation with the samples with and without RBD-SBD when compared to the control group (Figure S4). Therefore, these results confirm a dose-time-dependent PHB accumulation in the MRC5 cells and corroborate the cytotoxicity data by suggesting increased MP uptake without distinction between decorated and bare MPs.

The MP uptake was also evaluated by flow cytometry, which revealed a similar profile as previously shown by MTT and confocal analysis (Figure S5). Both sample groups are internalized without a statistical difference, indicating that the RBD domain causes no impact along the internalization process. Moreover, no difference was observed by expressing the RBD-SBD in HEK293 F cells to ensure a full glycosylation pattern, which displayed a similar uptake compared to bare and MP decorated with RBD-SBD produced in *E. coli*.

As MRC5 possesses a very low and basal ACE2 expression, we hypothesized that the internalization of MP containing RBD-SBD might increase ACE2 production as previously shown^45^ in EA.hy926 cells during a 72 h exposure up to 10 μg mL^–1^ of RBD. However, RT-qPCR and Western blot analysis could not detect the presence of ACE2 protein in the MRC5 cell samples after 72 h of continuous incubation with the samples (Figure S6). Altogether, these results highlight that MP internalization is mediated by unspecific uptake mechanisms that are not associated with intracellular signaling pathways elicited by a specific ligand–receptor interaction, followed by endocytic mechanisms. Moreover, they suggest limited MP trafficking across the membrane, probably as a result of the (i) micrometer-size range, (ii) rigid microrheological behavior and consequent inability to fuse with the phospholipid boundary as well as (iii) subthreshold protein decoration, and (iv) lack of segmental flexibility between the protein subunits for optimal ACE2 interaction. We intend to closely evaluate all of these particular features in the future to achieve a new generation of ligand-mediated nanodevices.

## 4 Conclusions

In this study, we highlight the effect of the SBD domain on sfGFP, mRFP1, and RBD immobilization on the surface of PHB. The PHB surfaces, MP or 2D films, that were incubated with the proteins linked to SBD displayed higher protein contents after the rising step, evidencing that SBD is essential for effective and irreversible anchoring. The PHB MP exhibited limited MRC5 internalization and cytotoxicity without a significant impact caused by the RBD protein, probably due to unspecific uptake induced by the micrometer-size range of the samples. The reported biotechnological strategy and anchoring process might be adapted for nanometric formulations specifically designed to interact with molecular targets. In particular, we intend to utilize similar bioinspired proteins to develop a new generation of nanovaccines with the potential to provide a fast-producing, interchangeable, and multivalent platform against several infectious diseases.

## Supporting Information Available

Full characterization of all proteins, including SDS-PAGE and Bradford quantification assay. More detailed data from CLSM, flow cytometry, and Western blot are also presented (PDF)
