# Supplementary material for: Controlling Protein Immobilization over Poly(3-hydroxybutyrate) Microparticles Using Substrate Binding Domain from PHA Depolymerase
Source: Biomacromolecules. 2025 Mar 10;26(4):2529–39. doi: 10.1021/acs.biomac.5c00010 (PMC12004514; doi:10.1021/acs.biomac.5c00010)
Supplement: Supplementary file 1 — bm5c00010_si_001.pdf [file bm5c00010_si_001.pdf]

Controlling protein immobilization over poly(3-hydroxybutyric acid) microparticles using substrate binding domain from PHA-depolymerase

Isabela P. Dias[1], Regiane Stafim da Cunha[2], Ryu Masaki[1], Maritza A. Todo Bom[1], Edneia A. S. Ramos[2], Giovanna Jardim[3], Giovanna Furman[1], Julia T. Lucena[1], Isabella G. Jiacomini[2], Sze M. Lo[2], Zelinda Schemczssen-Graeff[2], Breno C. B. Beirão[2], Silvio M. Zanata[2], Luiz M. de L. Faria[4], Edileusa M. Gerhardt[1], Emanuel Maltempi de Souza[1], Marcelo Müller-Santos[1], Guilherme F. Picheth[1,*]

[1]Department of Biochemistry, Federal University of Paraná, Curitiba, PR, Brazil.

[2]Department of Basic Pathology, Federal University of Paraná, Curitiba, PR, Brazil.

[3]Department of Genetics, Evolution, Microbiology and Immunology, University of Campinas, Campinas, SP, Brazil

[4]Department of Chemistry and Biology, Federal Technological University of Paraná, Curitiba, PR, Brazil.

* Corresponding author: Guilherme F. Picheth (gfpicheth@ufpr.br)

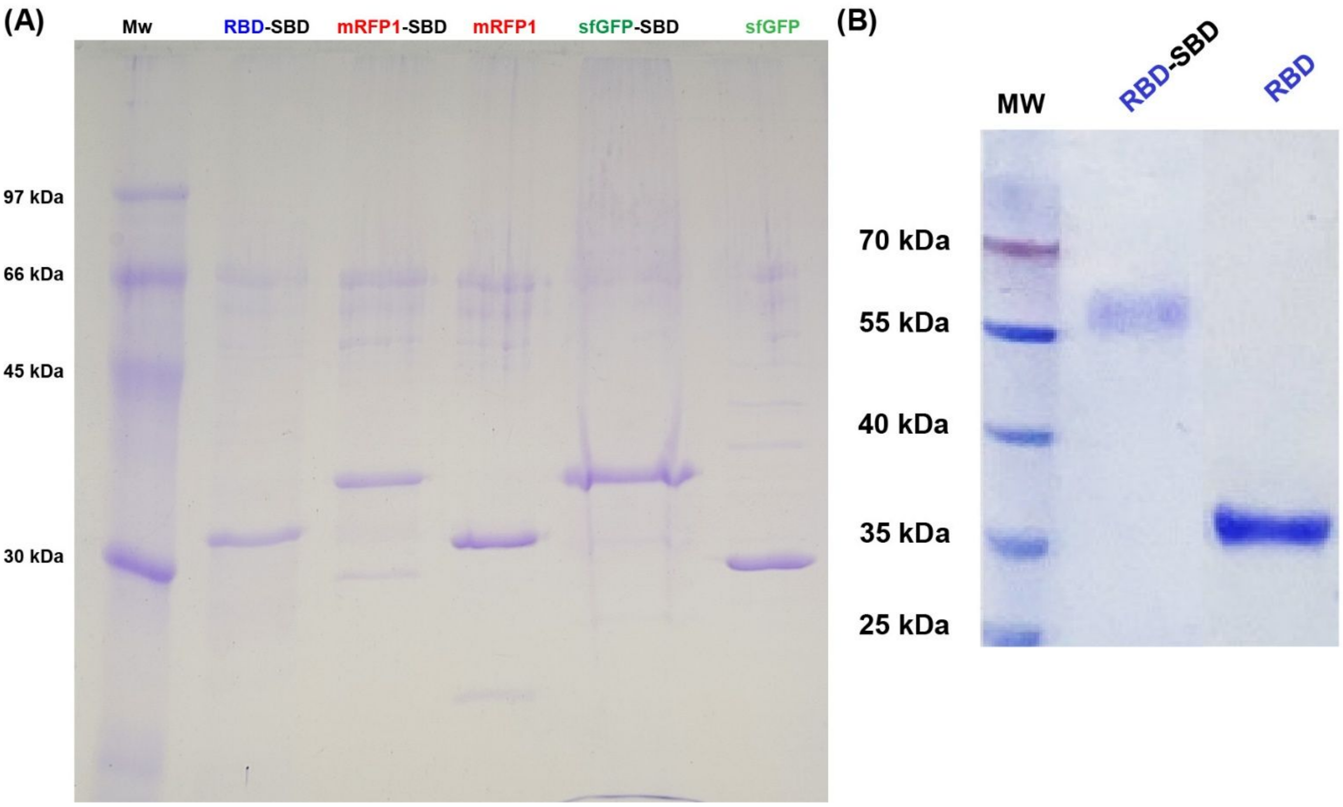

Figure S1. SDS-PAGE electrophoretic profile of all proteins expressed in *E. coli* (A) and HEK293 F (B) stained with Coomassie blue.

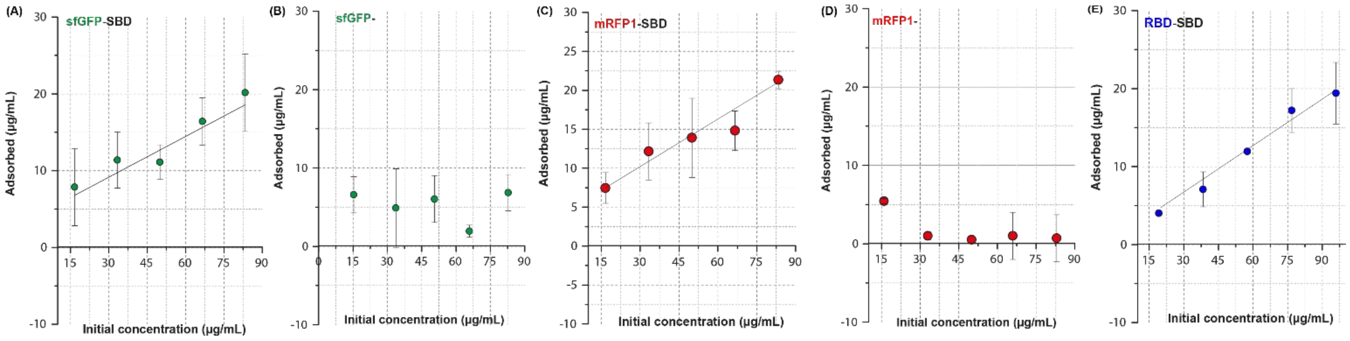

Figure S2. Protein adsorption quantification over the microparticles via Bradford for sfGFP-SBD (A), sfGFP- (B), mRFP1-SBD (C), mRFP1- (D) and RBD-SBD (E).

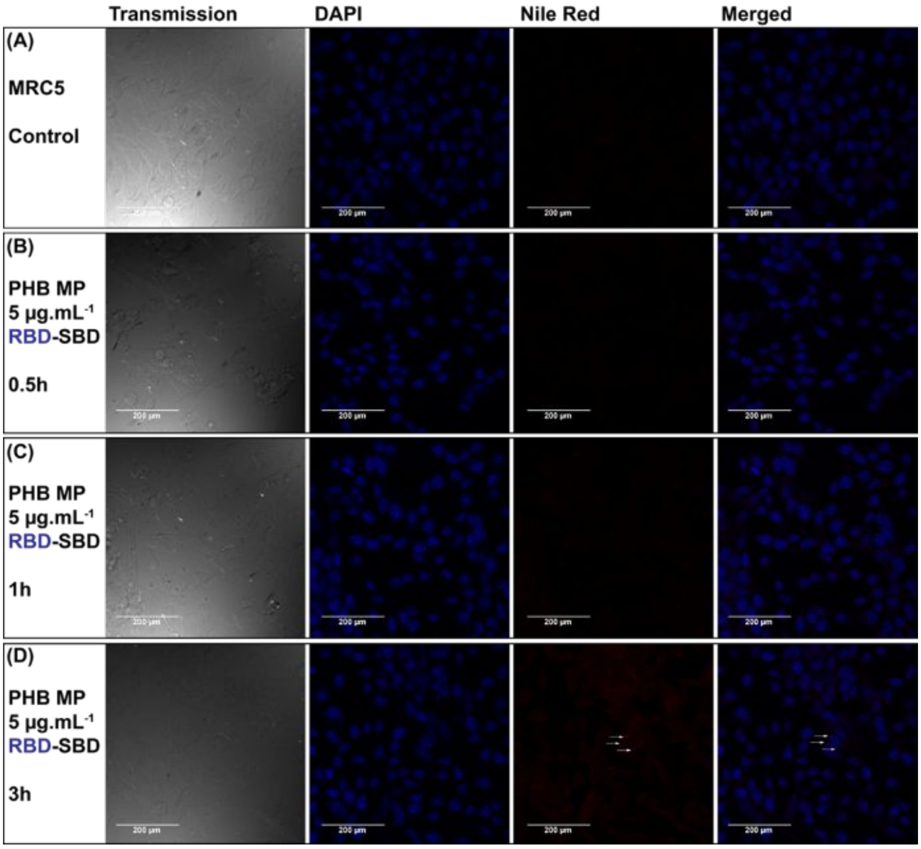

Figure S3. CLSM images of MRC5 cells incubated with MP (5 µg.mL^{-1}) decorated with RBD-SBD in different time intervals.

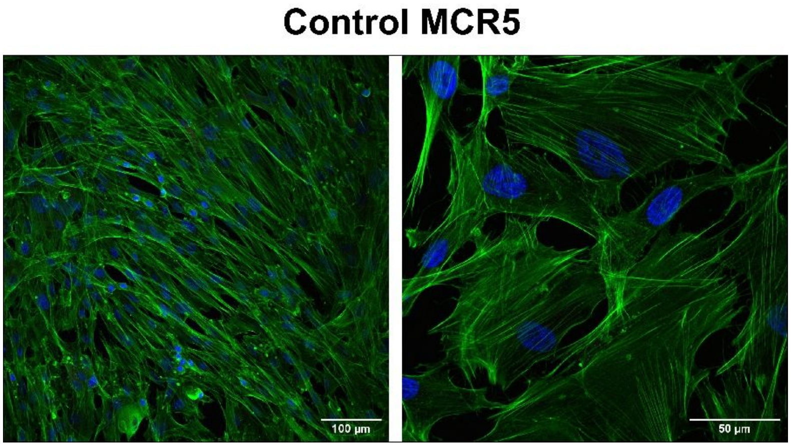

Figure S4. CLSM images of MRC5 cells control group, without MP incubation.

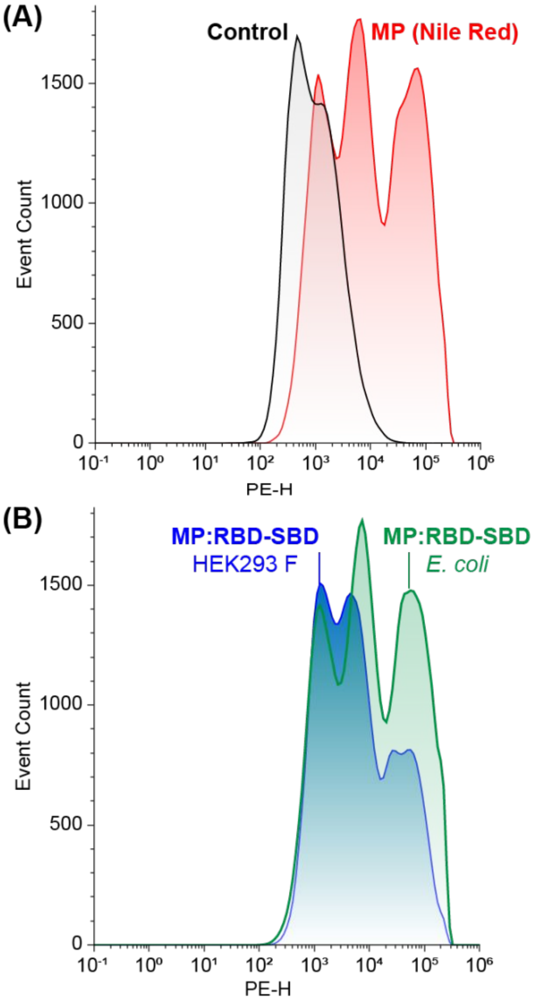

Figure S5. Flow cytometry analysis of MRC5 cells incubated with bare MP (A) and MP with RBD-SBD expressed in E. coli or HEK293 F (B) after 24h.

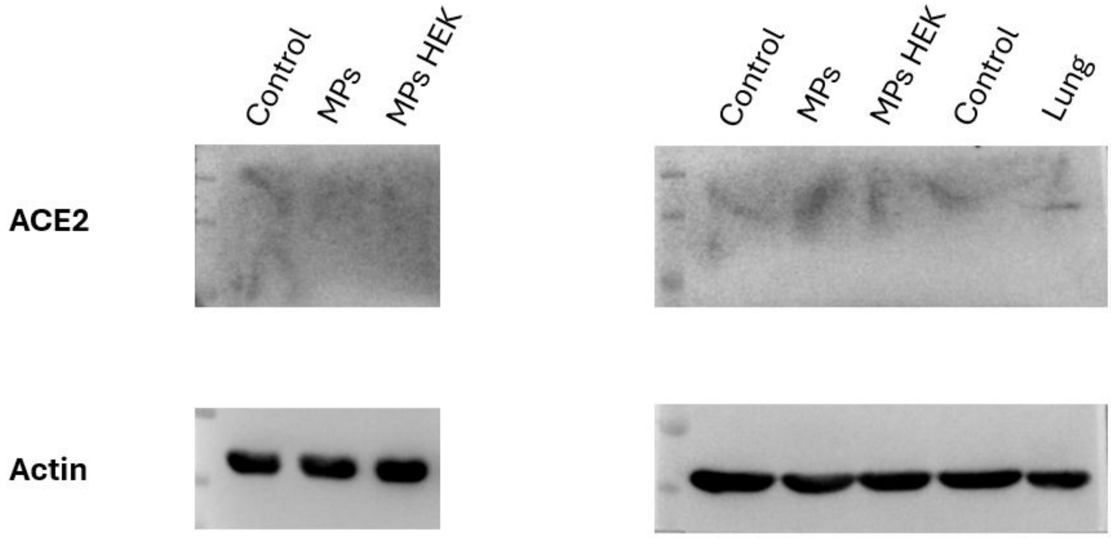

Figure S6. Western blot analysis of MRC5 ACE2 expression after 72h of continuous exposure to the samples.
